# Evaluating remote facilitation intensity for multi-national translation of nurse-initiated stroke protocols (QASC Australasia): a protocol for a cluster randomised controlled trial

**DOI:** 10.1186/s13012-023-01260-9

**Published:** 2023-01-26

**Authors:** O. Fasugba, S. Dale, E. McInnes, D. A. Cadilhac, M. Noetel, K. Coughlan, B. McElduff, J. Kim, T. Langley, N. W. Cheung, K. Hill, V. Pollnow, K. Page, E. Sanjuan Menendez, E. Neal, S. Griffith, L. J. Christie, J. Slark, A. Ranta, C. Levi, J. M. Grimshaw, S. Middleton

**Affiliations:** 1 Nursing Research Institute, St Vincent’s Health Network Sydney & St Vincent’s Hospital Melbourne & Australian Catholic University, Level 5, deLacy Building, St. Vincent’s Hospital, 390 Victoria Street, Darlinghurst, NSW 2010 Australia; 2 School of Nursing, Midwifery and Paramedicine, Australian Catholic University, Sydney, Australia; 3 Stroke and Ageing Research, Department of Medicine, School of Clinical Sciences at Monash Health, Monash University, Melbourne, Victoria Australia; 4 School of Psychology, University of Queensland, Brisbane, Australia; 5 St Vincent’s Health Network Sydney, Sydney, New South Wales Australia; 6 Centre for Diabetes and Endocrinology Research, Westmead Hospital and University of Sydney, Sydney, New South Wales Australia; 7 Stroke Foundation, Sydney, New South Wales Australia; 8 Vall d’Hebron Hospital Universitari Barcelona, Barcelona, Spain; 9 Prince of Wales Hospital, Randwick, New South Wales Australia; 10 Allied Health Research Unit, St Vincent’s Health Network, Sydney, Australia; 11 School of Allied Health, Faculty of Health Sciences, Australian Catholic University, Sydney, Australia; 12 School of Nursing, Faculty of Medical and Health Sciences, The University of Auckland, Auckland, New Zealand; 13 Department of Medicine, University of Otago Wellington, Wellington, New Zealand; 14 Department of Neurology, Wellington Hospital, Wellington, New Zealand; 15 John Hunter Health and Innovation Precinct, New Lambton Heights, New South Wales Australia; 16 Department of Medicine, University of Newcastle, Newcastle, New South Wales Australia; 17 University of Ottawa, Ottawa, Ontario Canada; 18 Ottawa Hospital Research Institute, Ottawa, Canada

**Keywords:** Stroke, Facilitation, Implementation, Remote, Process evaluation, Care bundle, Economic evaluation

## Abstract

**Background:**

Facilitated implementation of nurse-initiated protocols to manage fever, hyperglycaemia (sugar) and swallowing difficulties (FeSS Protocols) in 19 Australian stroke units resulted in reduced death and dependency for stroke patients. However, a significant gap remains in translating this evidence-based care bundle protocol into standard practice in Australia and New Zealand. Facilitation is a key component for increasing implementation. However, its contribution to evidence translation initiatives requires further investigation. We aim to evaluate two levels of intensity of external remote facilitation as part of a multifaceted intervention to improve FeSS Protocol uptake and quality of care for patients with stroke in Australian and New Zealand acute care hospitals.

**Methods:**

A three-arm cluster randomised controlled trial with a process evaluation and economic evaluation. Australian and New Zealand hospitals with a stroke unit or service will be recruited and randomised in blocks of five to one of the three study arms—high- or low-intensity external remote facilitation or a no facilitation control group—in a 2:2:1 ratio. The multicomponent implementation strategy will incorporate implementation science frameworks (Theoretical Domains Framework, Capability, Opportunity, Motivation – Behaviour Model and the Consolidated Framework for Implementation Research) and include an online education package, audit and feedback reports, local clinical champions, barrier and enabler assessments, action plans, reminders and external remote facilitation. The primary outcome is implementation effectiveness using a composite measure comprising six monitoring and treatment elements of the FeSS Protocols. Secondary outcome measures are as follows: composite outcome of adherence to each of the combined monitoring and treatment elements for (i) fever (*n*=5); (ii) hyperglycaemia (*n*=6); and (iii) swallowing protocols (*n*=7); adherence to the individual elements that make up each of these protocols; comparison for composite outcomes between (i) metropolitan and rural/remote hospitals; and (ii) stroke units and stroke services. A process evaluation will examine contextual factors influencing intervention uptake. An economic evaluation will describe cost differences relative to each intervention and study outcomes.

**Discussion:**

We will generate new evidence on the most effective facilitation intensity to support implementation of nurse-initiated stroke protocols nationwide, reducing geographical barriers for those in rural and remote areas.

**Trial registration:**

ACTRN12622000028707. Registered 14 January, 2022.

**Supplementary Information:**

The online version contains supplementary material available at 10.1186/s13012-023-01260-9.

Contributions to the literature

- This study will test whether high- or low-intensity external remote facilitation versus no external facilitation is more effective at improving implementation of the Fever Sugar Swallow (FeSS) Protocols for stroke patients in Australia and New Zealand.
- This study leverages a cluster randomized trial design, implementation science frameworks, a process evaluation and economic evaluation to enhance understanding of facilitation, and its contribution to implementation of evidence-based stroke interventions.
- This study aims to provide evidence on the most effective facilitation intensity for large-scale implementation of the FeSS Protocols in stroke patients.
- Findings will be relevant to FeSS Protocol adoption worldwide.

## Background

Facilitated implementation of a nurse-led intervention to manage *Fe*ver, hyperglycaemia (‘*S*ugar’) and *S*wallowing (FeSS Protocols) in 19 stroke units in New South Wales (NSW), Australia resulted in a 16% reduction in death and disability for patients with stroke in the Quality in Acute Stroke Care (QASC) Trial [1], with a sustained 20% improvement in survival 4 years post-stroke [2]. Subsequent scale-up of the Protocols to all 36 NSW stroke services in 2014 demonstrated improvements in Protocol adherence in the Quality in Acute Stroke Care Implementation Project (QASCIP) [3]. No further systematic implementation of the FeSS Protocols was initiated across Australia. To date, systematic implementation of the FeSS Protocols has not occurred in New Zealand.

In 2017, a ‘strong recommendation’ for use of standardised protocols to manage fever, glucose and swallowing difficulties in patients following acute stroke was included in the Australian and New Zealand Clinical Guidelines for Stroke Management [4]. However, the most recent 2021 Australian national acute services stroke audit showed that fever, hyperglycaemia and swallow management remained sub-optimal with only half (50%) of patients with fever ≥37.5°C receiving paracetamol within an hour, only 29% receiving insulin within 1 h of the first elevated finger prick glucose ≥10mmol/L and only 60% of patients receiving a swallow screen/assessment prior to oral medications, food, or drink [5]. In New Zealand, the most recent nationwide stroke audit in 2018 was conducted as part of the Reducing Ethnic and Geographic Inequities to Optimise New Zealand Stroke Care (REGIONS Care) Study. The findings showed that only 34.7% of ischemic stroke patients in urban hospitals and 32.5% of those in non-urban hospitals received a swallow assessment within 6 h of hospital arrival, with no significant difference between both groups (adjusted odds ratio 0.94, 95% confidence interval 0.74–1.18) [6]. Management of the other two FeSS Protocol elements (fever and hyperglycaemia) were not included in the audit hence it remains unclear whether or not these physiological parameters are being monitored and treated appropriately in New Zealand. The Australian and New Zealand audit results highlight that a significant evidence-practice gap remains in translation of the FeSS Protocols into standard stroke care across these countries. Achieving knowledge translation and sustaining adherence to evidence-based interventions is often difficult [7]. There is a need for systematic support to further improve adherence to the FeSS Protocols.

Successful implementation of research evidence into practice is influenced by the nature and type of evidence, the context in which the evidence is used, and use of an evidence-based implementation strategy [8, 9]. In particular, the need for appropriate facilitation as a strategy to improve the potential of implementation success has been emphasised [8]. Facilitation refers to the process of providing help and support to individuals and teams to enable them to achieve a specific goal by changing their behaviours [10]. Facilitation may occur in various forms, using either an external facilitator, an internal facilitator, or a combination of both [10]. Key roles of facilitators are to identify, engage and connect stakeholders; facilitate collaboration including the development of action plans; support communication and information sharing; and evaluate practice change [11]. Facilitator roles that have been examined in stroke include external facilitators providing ongoing remote or face-to-face support to clinicians to review hospital performance against clinical processes of care and develop an action plan [12, 13] and internal facilitators coordinating improvements in the organisation and delivery of stroke care [14, 15]. Evidence for the effectiveness of facilitation in the implementation of evidence into practice has also been demonstrated. Findings from a systematic review and meta-analysis showed a moderately robust effect for evidence-based guideline adoption following the use of practice facilitation [16]. A recent multicentre stepped-wedge cluster randomised trial demonstrated that externally facilitated educational outreach support aligned with routine collection of registry data, local consensus processes and tailored strategies has the potential to improve acute stroke care [17].

Our team have recently completed the QASC Europe project, a collaboration between the Nursing Research Institute, the European Stroke Organisation, the European Registry of Stroke Care Quality (RES-Q) and the European Acute Networks Striving for Excellence in Stroke (Angels) Initiative—a not-for-profit organisation that aims to optimise the quality of treatment in all stroke centres. This study provided further evidence for successful external remote facilitation resulting in international uptake of the FeSS Protocols into 67 European hospitals in 17 countries [18–20]. The results showed statistically significant improvement pre-to-post-implementation in overall FeSS Protocol adherence (Pre 3.4%, Post 35%; Absolute difference 33%, 95% CI 24%, 42%; *p*<0.0001 )[17]. In this pre-test/post-test study conducted amid the COVID pandemic, we used an external facilitation model whereby hospital clinical champions, supported by the Angels Initiative, implemented the FeSS Protocols with ongoing email and telephone support co-ordinated remotely from Australia. Consequently, the intervention was able to be successfully delivered simultaneously to multiple international stakeholders across Europe. The various stakeholder groups played different roles involving a flow of information and change management from one stakeholder level to another. We called this a Cascading Facilitation model which involved interlocking partnerships and supported facilitation [21].

There are many ways of providing facilitation which vary in their intensity, and level of resources required [10–15]. However, there is relatively little information about the effectiveness and cost-effectiveness of different facilitation models [13, 22, 23]. In particular, it is important to evaluate low-intensity/resource and high-intensity/resource approaches to identify effective and affordable facilitation models. Building on our previous research, this study will develop and test two different intensities of external remote (no face-to-face hospital visits by the research team) facilitation to support delivery of the FeSS Protocols for patients with stroke, at pace and on a large scale in two countries. Implementation of the FeSS Protocols will be informed by an integrated knowledge translation model [24]. This model incorporates evidence-based behaviour change implementation strategies (an online education package, audit and feedback reports, local clinical champions, barrier and enabler assessments, action plans, reminders and external remote facilitation) [25] and frameworks (Theoretical Domains Framework [TDF] [26], Capability, Opportunity, Motivation – Behaviour Model (COM-B system) [27] and the Consolidated Framework for Implementation Research [28]).

Cost-effectiveness studies evaluating the use of implementation strategies, including facilitation, in acute care settings are limited [29]. A study investigating changes in acute hospital costs before and after implementation of a stroke clinical facilitator programme found that the average cost per bed-day was higher post-implementation (AUD $878 per day) compared to pre-implementation (AUD $709). However, there was subsequent evidence of cost containment, with the average total inpatient costs per-episode decreasing by 10% over 4 years [30]. An independent economic evaluation estimated a gross economic benefit of AUD $281 million per year if the FeSS Protocols were implemented in 65% of the Australian stroke patient population [31]. A further study showed large societal and healthcare savings costs of AUD $251.7 million and AUD $65.2 million respectively for use of the FeSS Protocols over a 5-year period [32]. Consequently, we also will undertake a sub-study that includes an economic evaluation of the different facilitation strategies for implementing the FeSS Protocols.

A separate process evaluation will examine organisational, contextual and structural factors to successful uptake of the FeSS Protocols. Intervention fidelity will be examined as part of the main trial findings and reported as adherence with the FeSS Protocols as outlined in our outcome measures below.

### Study aims

The primary aim is to compare the effectiveness of high-intensity versus low-intensity external remote facilitation to support implementation of the FeSS Protocols to improve management of fever, hyperglycaemia and swallowing difficulties in the first 72 h of stroke unit/stroke service admission in Australia and New Zealand.

The secondary aims are to compare the effectiveness of (1) low-intensity facilitation with no facilitation, (2) high-intensity facilitation with no facilitation and (3) high- or low-intensity facilitation with no facilitation, to support implementation of the FeSS Protocols; (4) determine whether post-intervention changes in monitoring and treatment for fever, hyperglycaemia and swallowing differ between metropolitan and rural/remote hospitals; (5) determine whether post-intervention changes in monitoring and treatment for fever, hyperglycaemia and swallowing differ between stroke units and stroke services; (6) describe the potential cost-effectiveness of the different facilitation methods (economic evaluation); and (7) identify clinicians’ views regarding factors that influence uptake of the FeSS Protocols in metropolitan and rural/remote hospitals (process evaluation).

## Methods

### Study design

A three-arm cluster randomised controlled trial with a theory-based process evaluation will be undertaken. Hospitals (clusters) will be randomised to the multicomponent implementation strategy delivered with high or low external remote facilitation or a no facilitation control group receiving only usual care (inactive control group).

This protocol complies with the Standard Protocol Items: Recommendations for Interventional Trials (SPIRIT) [33], CONSORT extension for cluster randomised trials [34] and Template for Intervention Description and Replication (TIDiER) [35] checklists (Additional files 1, 2 and 3).

### Study setting

Australian and New Zealand public and private hospitals with a stroke unit or stroke service.

### Eligibility and recruitment of participants

#### Hospitals

To be eligible to participate in the study, hospitals must:

1. Have a pre-existing stroke unit (organised care within a specific ward in a hospital by a multidisciplinary team who specialise in stroke management) [36] or a stroke service (no dedicated stroke unit but with integrated hospital stroke services based on agreed hospital service delineations) [3];
2. Have at least one staff member to fulfil the role of hospital clinical champion;
3. Nominate staff to undertake patient medical record audits pre- and post-implementation and enter data into the Australian Stroke Data Tool or New Zealand Stroke Register; and
4. Grant permission to the researchers to obtain patient 90-180 day follow-up data from the Australian Stroke Clinical Registry (AuSCR) and the New Zealand Stroke Register for the economic evaluation (for those hospitals contributing data to these Registries).

In Australia, eligible hospitals will be systematically recruited through invitations to all stroke units/stroke services known to the Stroke Foundation. The study will also be advertised through the AuSCR, Stroke Foundation, Australian Stroke Nurses Education Network and state-based stroke clinical networks via email, newsletters and websites. In New Zealand, recruitment will occur through the National Stroke Network, Stroke Nurse Forum Aotearoa and clinical networks. A short recruitment video will also be developed and shared to these organisations in advertisement emails and via social media.

#### Patients

The medical records of eligible patients will be audited by participating hospital staff. Patients will be eligible if they are aged 18 years or older and have a diagnosis of ischaemic stroke, intracerebral haemorrhage, or stroke of undetermined origin with the following International Classification of Diseases, Tenth Revision (ICD-10) codes: I61.0 - I61.9 (intracerebral haemorrhage), I63.0 - I63.9 (cerebral infarction), I64 (stroke not specified as haemorrhagic or infarction) and I62.9 (intracranial haemorrhage unspecified) [37]. Patients will be excluded if they are admitted for palliative care.

### Randomisation and blinding

**Fig. 1:**
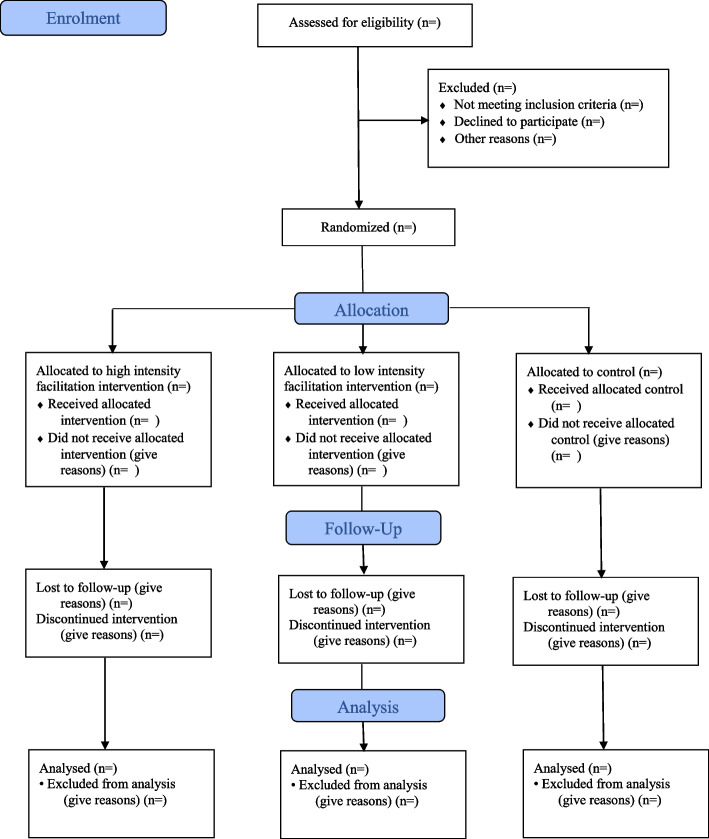
CONSORT flow diagram

The unit of randomisation will be hospitals. Hospitals will be randomised in blocks of five to one of the three study arms—high- or low-intensity external remote facilitation or a no facilitation control group—in a 2 (high intensity):2 (low intensity):1 (no facilitation) ratio. Prior to randomisation, hospitals will be stratified by country, participation in a stroke registry and remoteness area factor (metropolitan/major cities or regional/rural as per the Accessibility and Remoteness Index of Australia [ARIA+] [38] and urban or non-urban as per the New Zealand REGIONS Care project [39] classification). Hospitals that meet the inclusion criteria will be enrolled on a rolling basis until the target sample size is reached. A flow diagram outlining the randomisation and intervention allocation is shown in Fig. 1.

Computer-generated randomisation of the hospitals will be performed independently by a statistician blinded to group allocation and not otherwise involved in the study ensuring allocation concealment. Staff at participating hospitals will be blinded to the intensity (high, low or none) of external remote facilitation provided.

### Intervention

The QASC Australasia intervention is a multicomponent implementation strategy aimed at changing clinician and team behaviour [26, 27]. Multidisciplinary stroke unit/stroke service staff will be required to work together to achieve the study outcomes. The intervention components, mapped to several Theoretical Frameworks (Additional file 4), are outlined below and summarised in Table 1.

**Table 1:** Components of QASC Australia Intervention

Components	Intervention groups		Control group
	High-intensity facilitation	Low-intensity facilitation	No facilitation
FeSS Protocols	✓	✓	✓
Online FeSS education package	✓	✓	X
Evidence-based audit and feedback report	✓	✓	X
External remote facilitation:			
*Videoconference sessions for timeline, action plan development and implementation*	✓	X	X
*Reminders*	Project process, intervention and data collection reminders	Project process, intervention and data collection reminders	Project process and data collection reminders only
*Email and telephone support*	Proactive and reactive	Reactive	X

#### FeSS protocols

The evidence-based care bundle of FeSS Protocols (Table 2) refined from our previous FeSS implementation studies [1, 2] will be used in this national translational study.

**Table 2:** Summarised elements of the Fever, Hyperglycaemia (Sugar) Swallow (FeSS) Protocols

**Fever (*n*=2)** • Temperature readings monitored and recorded at least four times per day for the first 72 h • If temperature => 37.5°C treat with paracetamol or other anti-pyretic **Sugar (Hyperglycaemia) (*n*=3)** • Formal venous glucose on admission to Emergency Department or stroke service • Blood glucose level readings monitored and recorded at least four times per day for the first 48 h, to continue for 72 h if BGL unstable • If blood glucose level >10 mmol/L (180mg/dl) treat with insulin **Swallowing (*n*=2)** • Swallow screen or swallow assessment within 4 h of admission and prior to being given oral food, drink, or medications • Referral to speech pathologist for full assessment for those who fail the swallow screen	

#### Implementation strategy

All intervention components (FeSS Protocols, online FeSS education package, FeSS audit and feedback report) including external remote facilitation will be provided online. Participating hospitals will nominate at least one clinician (nurse where possible, or if not, then a speech pathologist) currently working in the stroke unit/stroke service to act as a hospital clinical champion. Preferably, this would be a senior clinician(s) with stroke expertise, but more importantly, someone recognised as a stroke clinical leader within their hospital, ready to implement change at the hospital and encourage use of the FeSS Protocols by their local team. Evidence suggests that key attributes of a clinical champion which have the potential to improve implementation outcomes are influence, ownership, physical presence at the point of change, grit, persuasiveness and participative leadership style [40]. Our team has had considerable success in NSW and in Europe working with clinical champions to support evidence translation locally [1, 3, 18]. The evidence-based implementation strategy comprises an educational package [41], audit and feedback reports [42], local clinical champions [43], barrier and enabler assessments [44, 45], action plans [25], reminders [46] and external remote facilitation [12] (Table 1).

#### Online FeSS education package

Education and training of stroke clinicians is important in ensuring the delivery of evidence-based stroke care and improvement in outcomes for patients with stroke [47]. The use of information and communication technologies in the education of clinicians is now common globally. E-learning environments integrate information, in the form of text and multimedia, and can be designed for self-directed learning or learning in real time [48]. This delivery method for learning has gained popularity due to the potential benefits of increasing the accessibility of information to remote learners, lower costs, personalised instruction in terms of content and pace of learning and facilitating frequent content updates [49].

The online FeSS education package consists of three components: An Online Education Resource Package; a Train-the-Trainer Education Package; and a Clinician Education Package. All components of the package will be provided to clinical champions in intervention hospitals. Each component is outlined in Table 3 and described further below:

**Table 3:** Components of the FeSS education package

**Online Education Resource Package** • Videos with the following topics: o FeSS is one of the most cost-effective interventions in acute stroke care o A fever is not the smoke, it’s the fire. Put it out ASAP o Blood sugar is the fuel on the fire: keep it regulated o A sip of water can be deadly after strokes: Check their swallowing first o Leading change can be hard. Here’s how to make it easier o Why do an audit? o How to do an audit • Downloadable hard-copy resources o FeSS Protocols o PowerPoint slides for multidisciplinary meeting o PowerPoint slides for education session o Action plan template o PowerPoint slides for ASSIST education o PowerPoint slides for ASSIST competency assessment o QASC Australia data dictionary o QASC Australia data collection user manual o Flowchart of project milestones o Frequently asked questions (FAQ) document	
**Train-the-Trainer Education Package** (relevant to study group allocation) • High- and low-intensity external remote facilitation groups o All components of the online education resource package • Control group will receive only downloadable hard-copy resources o FeSS Protocols o PowerPoint slides for ASSIST education o PowerPoint slides for ASSIST competency assessment o QASC Australia data dictionary o QASC Australia data collection user manual o Frequently asked questions (FAQ) document	
**Clinician Education Package** (high- and low-intensity external remote facilitation groups only) • Short (5-7 minute) videos with the following topics: o FeSS is one of the most cost-effective interventions in acute stroke care o A fever is not the smoke, it's the fire. Put it out ASAP o Blood sugar is the fuel on the fire: keep it regulated o A sip of water can be deadly after strokes: Check their swallowing first o Why do an audit? o How to do an audit • Downloadable hard-copy resources o FeSS Protocols	

##### Online Education Resource Package

An online education resource package comprising a suite of seven videos of 5–7-min duration and downloadable hard-copy resources will be developed by the research team in collaboration with educational psychologists specialised in adult learning research from the School of Psychology, University of Queensland. Video topics will be based on case studies using stroke experts, consumers, role-playing actors and animations. Learning activities will be included at the end of each video with summative case studies to assess participants knowledge following completion of the videos. The package will be hosted on the Australian Catholic University OpenLearning webpage. The design of the videos will incorporate implementation science behaviour change techniques which will be mapped to behaviour change frameworks and taxonomy (v1), COM-B and TDF [27, 50, 51] (Additional file 5) to provide clinicians with the capability, opportunity and motivation to implement the FeSS Protocols. In addition, the online video package will be designed using effective multimedia principles to optimise learning in viewers by leveraging the two key information processing systems—sight and sound, and managing cognitive load [52, 53]. Designs were informed by adult learning principles previously developed and evaluated by members of our team who developed and implemented a suite of educational resources. These resourcses successfully changed behaviour in primary school teachers to increase physical activity in children [54–57].

##### Train-the-Trainer Education Package

Clinical champions will be encouraged to form their own local implementation team consisting of themselves and at least a medical doctor, and a speech pathologist, from the stroke unit/stroke service with expertise in acute stroke patient management. The clinical champions will receive all components of the online FeSS education package relevant to their study group allocation. Clinical champions will have continued access to the online FeSS education package throughout the duration of the project to support them in implementing the FeSS Protocols.

##### Clinician Education Package

Clinical champions will be responsible for educating all clinicians (nurses, doctors and speech pathologists) in their stroke units/stoke services. They will run the on-site multidisciplinary education session multiple times to reach the majority of clinicians. Clinical champions will use the online education resources to explain to stroke unit/stroke service clinicians about the seven monitoring and treatment elements of the FeSS Protocols (Table 2) and implementation of the Protocols. A detailed explanation of the research evidence underpinning the FeSS Protocols including the rationale for its inclusion in the stroke guidelines will also be provided at the session. Clinical champions will be permitted to share modules from the online education package with the clinicians who are unable to attend the session and arrange make-up training.

#### FeSS audit and feedback report

Clinical champions in intervention hospitals will receive a confidential online baseline report of their pre-implementation audit data on FeSS Protocol compliance prior to the intervention. They will be provided with one post-implementation audit report about 2 weeks following completion of the post-implementation audit. Intervention group hospitals will receive detailed reports which provide information on the implications of the findings as well as recommendations for improvement. This report is designed to meet the fifteen recommendations believed to be associated with effective audit and feedback interventions [58]. We used this report in our previous QASC Europe project [17, 20], and after obtaining end-user feedback, it was refined for use in the current study. We have also successfully pilot tested the report with stroke nurse experts.

#### External remote facilitation

Three researchers with health qualifications and expertise in stroke research will provide external online only (i.e. no site visits) remote facilitation to intervention hospitals. Their role will involve providing information about the study to participating hospitals and granting intervention hospitals access to the online education package. They will guide clinical champions in the high-intensity external remote facilitation intervention group in undertaking self-directed training to develop their capability and capacity in leading and negotiating evidence-based change. Clinical champions in the low-intensity external remote facilitation intervention group will not receive guidance from the external facilitators but will be provided with the flowchart of project milestones to ensure they undertake the audit training within the specified timeframe. The external facilitators will also support intervention hospitals in the high-intensity external remote facilitation group via videoconference sessions (described below; Table 4) but will not be involved in data collection.

**Table 4:** Time points for 1-h external facilitation videoconference sessions

• Post-randomisation - To discuss pre-implementation audit results and the online FeSS education package • After clinical champions receive the online FeSS education package - To discuss progress with completing the package • After clinical champions conduct the multidisciplinary meeting - To assist with action plan development • At commencement of 3-month bedding down period - To discuss any issues with FeSS Protocol implementation	

#### Videoconference sessions

The three external facilitators will lead the videoconference sessions, comprised of a didactic component using standardised PowerPoint slides, and inter-active group participation. These sessions will be target clinical champions in the high-intensity external remote facilitation intervention group. Sessions will be delivered in groups and the number of clinical champions in a group will depend on their availability to attend a scheduled session. A minimum of four, 1-h videoconference sessions are planned (Table 4). These will be held at key project milestones, specifically post-randomisation (to discuss pre-implementation audit results and the online FeSS education package); after receiving the online FeSS education package (to discuss progress with completing the package); after the multidisciplinary meeting (to assist with action plan development); and at commencement of the 3-month bedding down period (to discuss any issues with FeSS Protocol implementation). Sessions will be repeated as many times as necessary to ensure all clinical champions receive the same high-intensity dose of external remote facilitation. Any additional sessions and their timing will be determined by demand from the clinical champions group.

#### Local team meeting to assess barriers and develop action plan

Clinical champions in intervention hospitals will be provided with resources from the online FeSS education package to conduct a local multidisciplinary meeting to identify barriers and enablers to the FeSS Protocol uptake. A detailed baseline pre-implementation audit and feedback report of FeSS processes of care will be presented by the clinical champion(s) at the meeting as the evidence base for current standard of care for stroke patients at the hospital. The meeting will be used by stroke unit/stroke service teams to work together to identify potential barriers and enablers to implementation of the FeSS Protocols and develop an Action Plan with local solutions. The clinical champions will be provided with an Action plan template previously piloted in the QASC and T^3^ Trials [1, 59], and developed using the model suggested by French and colleagues [50]. This Action plan will be sent to the researchers at the Nursing Research Institute and will be used to guide the external remote facilitation videoconference sessions held by the researchers for intervention hospitals in the high-intensity external remote facilitation intervention group.

#### Reminders

The high and low facilitation intervention group hospitals will be sent intervention reminders (Table 5) to prompt them to progress with FeSS Protocol implementation.

**Table 5:** Timepoints and details of reminders

Milestone	First email	Second email	Third email^**a**^
***Project process reminders (high- and low-intensity intervention group and control group hospitals)***			
EOI received to participate in study	Email to sign Agreement Form, nominate clinical champion(s), complete organisational survey online and assist with obtaining site-specific governance approval	Two weeks later same email if no response	Two weeks later same email if no response
Site-specific governance approval received	Confirmation email sent		
Data collection reminders (high- and low-intensity intervention group and control group hospitals)^*^			
Pre-implementation audit	Email sent 1 week before pre-implementation audit date (3 months post-study commencement) with data entry instructions	Email sent 2 weeks after pre-implementation audit date if no data entry	Email sent 4 weeks after pre-implementation audit date if no data entry
Completion of pre-implementation audit and data queries from statistician	Confirmation email sent with any requests for data checks (if required) based on preliminary data cleaning 1 week after completion of audit		
Reliability cases sent to hospitals for data checks	Email sent requesting data reliability checks 1 week after completion of audit	Email sent 2 weeks later if no response	Email sent 2 weeks later if no response
Pre-implementation audit and feedback report	Email sent with report 2 weeks after completion of audit		
Post-implementation audit	Email sent on post-implementation audit date (6 months post-implementation)	Email sent 4 weeks after post-implementation audit date if no data entry	Email sent 6 weeks after post-implementation audit date if no data entry
Completion of post-implementation audit and data queries from statistician	Confirmation email sent with any requests for data checks (if required) based on preliminary data cleaning 1 week after completion of audit		
Reliability cases sent to hospitals for data checks	Email sent requesting data reliability checks 1 week after completion of audit	Email sent 2 weeks later if no response	Email sent 2 weeks later if no response
Post-implementation audit and feedback report	Email sent with report 2 weeks after completion of audit		
***Intervention reminders (high-intensity and low-intensity intervention group hospitals only)***			
FeSS Protocol implementation	Email sent requesting multidisciplinary meeting date/s	Email sent 2 weeks later if no response	Email sent 2 weeks later if no response
Action plan	Email sent requesting action plan	Email sent 2 weeks later if no response	Email sent 2 weeks later if no response
FeSS Protocol implementation ‘go-live’ date	Email sent 1 month after meeting requesting ‘go-live’ date	Email sent 1 month later if no response	Email sent 2 weeks later if no response

^a^Further email prompts may be required; ^*^Control group hospitals do not receive emails with the audit and feedback report

#### Email and telephone support

The researchers will provide clinical champions from the intervention group hospitals with ongoing proactive and reactive email and telephone remote support (Table 6).

**Table 6:** Email and telephone external remote support^a^

***Email*** ▪ Proactive emails from research team to clinical champions during key milestones (post-randomisation; post-receipt of online FeSS education package; post-multidisciplinary meeting; commencement of 3-month bedding down period) ▪ Reactive emails from clinical champions to research team when required ***Telephone*** ▪ Proactive telephone contact from research team to clinical champions following completion of pre-implementation audit data collection ▪ Reactive telephone contact from clinical champions to research team when required	

^a^High-intensity external remote facilitation (proactive and reactive); low-intensity external remote facilitation (reactive only)

### Control group

Hospitals randomised to the control group will only have access to the publicly available FeSS Protocols. No active intervention/external remote facilitation will be provided by the researchers, and these hospitals will perform usual stroke care practices and collect data on the FeSS processes of care. Control group hospitals will not receive the detailed FeSS audit and feedback reports that will be provided to intervention group hospitals. However, there may be some hospitals randomised to the control group that participate in the biennial national stroke acute care audits and/or contribute continuous data on the FeSS elements to the AuSCR. As per standard practice, these hospitals will routinely receive basic audit and feedback reports from the Stroke Foundation and/or AuSCR as part of quality improvement initiatives undertaken external to the trial. If the research team receive any proactive emails from control group hospitals regarding the study (excluding questions about data collection), these hospitals will be directed to the Quality in Acute Stroke Care webpage hosted on the Australian Catholic University website (https://www.acu.edu.au/qasc). If the questions relate to the national stroke audit or AuSCR data entry or platform issues, they will be referred to the national stroke audit coordinator or AuSCR, as required. At study completion, clinical champions from control group hospitals will receive access to the online FeSS education package after providing their post-implementation data.

### Data collection

Pre- and post-implementation data will be entered by hospital staff into the web-based Australian Stroke Data Tool or directly into the New Zealand Stroke Registry, for Australian and New Zealand hospitals respectively. The Australian Stroke Data Tool is an integrated data management system used to collect patient data for the Stroke Foundation national audits and the Australian Stroke Clinical Registry [60]. Access to the data is password protected and the server has an effective firewall and security policies that are regularly reviewed and maintained to ensure adherence to all local and national privacy laws and principles. Medical notes of up to 100 eligible patients or 6 months of eligible stroke patient data will be retrospectively reviewed for the pre-implementation audit. For the post-implementation audit, hospital staff will be required to undertake medical record audits for 100 prospective consecutive stroke admissions or up to a period of 6 months, providing data for similar numbers of patients pre- and post-implementation. Demographic and clinical data to be obtained include the following: hospital name, patient medical record number, age, sex, date of stroke onset, date of admission, stroke type, stroke severity using the National Institutes of Health Stroke Scale [61], premorbid risk factors and functional status using the modified Rankin Scale score [62], treatment in a stroke unit, FeSS variables, discharge modified Rankin Scale and discharge destination.

Clinicians from participating hospitals will receive video instructions on how to undertake a medical record audit and enter data into the Australian Stroke Data Tool for uniformity in data collection processes. All participating hospitals will be sent project process and data collection email reminders by the research team at critical timepoints during the project (Table 5). Hospitals in the control group will undertake data collection at the same time as the intervention hospitals with which they are blocked randomised.

Participating hospitals will also be asked to complete a short online organisational survey to obtain information about structural workplace characteristics and resources available to deliver acute stroke care to allow for more accurate data comparison. Data to be collected include presence of stroke unit, bed capacity, availability of multidisciplinary and specialist staff, annual thrombolysis rate (previous 12 months) and presence of stroke care facilitators from the Angels Initiative (a not-for-profit organisation that aims to optimise the quality of treatment in stroke centres [https://www.angels-initiative.com/]).

All data will be stored, managed and archived in accordance with National Health and Medical Research Council requirements. Electronic data will be stored on password-protected networked drives linked to the Nursing Research Institute and accessible only by the researchers. The final trial data set will be archived in a data repository. Only de-identified data will be analysed. The study findings will be communicated via publication in peer-reviewed scientific journals.

### Study procedure

Following the recruitment process, stroke unit/stroke service coordinators of eligible hospitals who express interest in participating will be sent an email requesting them to sign an Agreement Form indicating hospital consent to participate. They will also be requested to nominate a hospital clinical champion who will complete the online organisational survey and assist with obtaining site-specific governance approval and act as the hospital liaison officer with the researchers. After receipt of this approval, hospitals will be required to complete the pre-implementation retrospective medical record audit following which they will be randomised to one of the two intervention or control groups. From the date of randomisation, the clinical champion(s) of intervention hospitals will be required to undertake the online FeSS education package training and organise a local multidisciplinary barriers and enablers identification meeting. We will request that this training and meeting be completed within 6 weeks of hospital randomisation; however, should this not occur, we will negotiate intervention start dates with these hospitals. Hospitals will then commence a 3-month implementation period which will involve clinical champions running the multidisciplinary barriers and enablers identification meeting, clinician education session and commence implementation of the FeSS Protocols. It is anticipated that the ‘go-live’ date (when the FeSS Protocols are considered normal business and are fully operational) will be scheduled for the end of the 3-month implementation period. Hospitals then will have a further 3 months ‘bedding down’ of the intervention for it to become routine practice [1] following which the post-implementation prospective medical records audit will be commenced. Further details about the trial enrolment, interventions and assessments are shown in Table 7.

**Table 7:** Schedule of enrolment, interventions and assessments

TIMEPOINT	Study period							
	Pre-intervention activities	Randomisation	Intervention			Post-intervention activities		Close-out/completion of trial
	Q4 2022	Q1 2023	Q2–Q4 2023			Q4 2023–Q3 2024		Q4 2024
**ENROLMENT:**	x	x						
Eligibility screen	x	x						
Informed Agreement	x	x						
HREC and Governance applications	x	x						
Baseline data collection	x	x						
Randomisation of hospitals		x						
**INTERVENTION**			**High facilitation**	**Low facilitation**	**Control**			
Baseline audit and feedback report			x	x				
Email FeSS Protocols					x			
Videoconference 1			x					
Email link to online FeSS education package			x	x				
Videoconference 2			x					
Multidisciplinary Team barrier assessment workshop (Action plan development)			x	x				
Videoconference 3			x					
Education sessions for stroke unit/stroke service clinicians			x	x				
Recruitment of clinicians for process evaluation interviews and focus groups			x	x	x			
Videoconference 4			x					
**3 months bedding down of FeSS Protocols**			x	x	x			
Post-intervention audit data collection			x	x	x	x		
Post-intervention audit and feedback report			x	x				
**ASSESSMENTS**								
Overall FeSS adherence composite measure	x					x		
Individual monitoring and treatment elements of the FeSS Protocols	x					x		
Death and dependency (mRS) *sub-study only*	x					x		
EQ-5D-3L questionnaire *sub-study only*	x					x		
Costs of implementing the FeSS Protocols	x	x	x	x	x	x	x	x
**Process evaluation interviews and focus groups**						x	x	
**Economic evaluation**						x	x	x

### Economic evaluation

An economic evaluation will include a cost-consequence analysis to evaluate the costs and benefits of the external remote facilitation (low and high) interventions, relative to control. We will also assess the potential cost-effectiveness of the interventions compared with control by estimating incremental cost-effectiveness ratios (ICERs) [63]. All costs will be presented based on the 1-year period of the QASC Australasia programme. Cost will be converted into Australian/New Zealand dollars using a nominal rate method and adjusted for inflation where necessary. The additional costs of implementing the FeSS Protocols will be informed by financial records kept by the researchers. Other additional costs or cost savings due to the implementation of the FeSS Protocols will be considered based on the findings of the clinical evaluation, the findings of the process evaluation, and consultation with the researchers.

Data on health benefits will be derived in a sub-study of this cluster randomised controlled trial. This sub-study will only include trial hospitals that contribute data to the AuSCR [64] or the New Zealand Stroke Registry because the registries collect 90–180-day patient outcome data required for the economic evaluation. For this subset of hospitals contributing FeSS data to the registry [64], patient hospital data collected in the Australian Stroke Data Tool together with the follow-up survey data collected between 90 and 180 days from the AuSCR will be used. Quality-adjusted life years (QALYs) will be estimated by applying utility scores according to the modified Rankin Scale at discharge from hospital, from data obtained at a follow-up assessment conducted at 90–180 days post-stroke as part of the AuSCR and survival status determined through routine linkage of the AuSCR with the National Death Index. Responses to the EQ-5D-3L questionnaire at 90–180 days post-stroke will be converted into a utility score using an algorithm developed in an Australian population [65].

Data from the study will be supplemented in the economic analysis models by applying estimates from the published literature on treatment effect, discharge destination and outcomes, as required. The economic evaluation will be reported according to the Consolidated Health Economic Evaluation Reporting Standards [66], and the findings may be used to model the potential benefits of FeSS implementation state-wide or Australia-wide.

### Process evaluation

Successful nationwide implementation of the FeSS Protocols depends on the social and cultural context and behaviours of those delivering or receiving the intervention. Nurses and doctors acceptance and capacity to fully adhere to the Protocols is also central to maintaining intervention fidelity [67]. Hence, study outcomes (i.e. intervention success or failure) may be affected by factors related to the implementation or delivery of an intervention. Therefore, evaluation of the processes related to intervention delivery is important to provide insight into why interventions work or fail and how they can be improved.

A qualitative descriptive design will be used for the process evaluation. Frameworks for the design and reporting of the process evaluation of complex interventions developed by the United Kingdom Medical Research Council [68] and cluster randomised controlled trials [69] will inform the design, conduct and reporting of the process evaluation. At the conclusion of the trial, clinical champions, stroke unit/stroke service medical and nursing heads and bedside nurses implementing the FeSS Protocols will be approached by members of the research team requesting voluntary participation in semi-structured interviews and focus groups. Interviews and focus groups will examine the contributing organisational, contextual and structural factors that impacted successful/unsuccessful uptake of the intervention. Clinicians will be purposively sampled from high- and low-intensity external remote facilitation intervention hospitals further stratified by level of adherence (greater than or less than median; resulting in four strata) to the FeSS Protocols based on final trial results. We will sample a minimum of two hospitals from each stratum (*n*=8 hospitals) from which we will purposively sample one clinical champion, one medical head and one nursing head for interviews (*n*=3 interviews per hospital) and one focus group of bedside nurses from each hospital. Hence, we will undertake approximately 24 semi-structured interviews and eight focus groups, depending on saturation. Provision will be made for interviews and focus groups to be conducted virtually via videoconferencing or in person depending on the COVID-19 pandemic.

The focus group and interview questions will be designed using the Normalisation Process Theory [70]. The Normalisation Process Theory identifies and explains key mechanisms that promote and hinder the implementation, embedding and integration of complex interventions [70]. Two weeks prior to the focus groups and interviews, an information letter with details of the study including the procedure for consent will be provided to participants by the research team. Focus group and interview questions will be mainly open-ended and sessions will be conducted by an independent researcher member of the research team who has expertise in multi-method process evaluations of clinician practice change. Focus groups and interviews will be audio recorded, with the permission of participants, for ease of transcription. No identifiable information provided during interviews will be linked to individual participants with all identifiers removed before analysis. Voice recordings from the focus groups/interviews will be permanently deleted once a transcript of voice recordings is performed. All study materials will be disposed of in a confidential manner by shredding all focus groups/interviews transcripts and erasing all audiotapes and computer files.

### Outcome measures

The primary outcome is a binary measure of defect-free care. Defect-free care [71] is defined as adherence to a composite measure of six monitoring and treatment elements of the FeSS Protocols (Table 8). We have chosen one monitoring and one treatment element each from the Fever and Sugar Protocols and two elements from the Swallow Protocol, to be collected on the day of admission to the stroke unit/stroke service. These six elements were chosen as they are thought to be a proxy for better Protocol adherence for stroke care provided on day 2 and day 3 of hospital admission.

**Table 8:** FeSS Protocol Outcome Measures

• ^b^*Fever Protocol* o **Temperature monitored at least four times per day on day of admission**^a^ o Temperature monitored at least four times per day on day 2 of admission o Temperature monitored at least four times per day on day 3 of admission o Paracetamol (or other antipyretic) given for first temperature ≥37.5°C o **Paracetamol (or other antipyretic) given with 1 h from first temperature ≥37.5°C**^a^ • ^b^*Hyperglycaemia (Sugar) Protocol* o Venous blood glucose level sample collected and sent to laboratory on admission to hospital o **Blood glucose levels (BGL) monitored at least four times per day on day of admission**^a^ o BGLs monitored at least four times per day on day 2 of admission o BGLs monitored at least four times per day on day 3 of admission (if BGLs unstable) o Insulin given for first BGL >10mmol/L o **Insulin given within 1 h from first BGL >10mmol/L**^a^ • ^b^*Swallow Protocol* o Formal swallow screen performed o Failed screen and subsequently had swallow assessment o Swallow screen performed within 4 h o **Swallow screen performed within 24 h**^a^ o Swallow assessment recorded o Swallow screen recorded o **Swallow screen or assessment performed before being given oral medications, food, or fluids**^a^	

^a^To be included in the primary composite outcome comprising six monitoring and treatment elements of the FeSS Protocols

^b^To be included in the secondary composite outcome of adherence to each of the combined monitoring and treatment elements for (i) fever, (ii) hyperglycaemia (sugar) and (iii) swallowing protocols

The secondary outcome measures are composite outcomes of adherence to each of the combined monitoring and treatment elements for (i) fever, (ii) hyperglycaemia (sugar) and (iii) swallowing protocols, and to the individual elements that make up each of these protocols (Table 8); comparison between metropolitan and rural/remote hospitals for composite outcome of adherence to each of the combined monitoring and treatment elements for (i) fever, (ii) hyperglycaemia (sugar) and (iii) swallowing protocols, and to the individual elements that make up each of these protocols; and comparison between stroke units and stroke services for composite outcome of adherence to each of the combined monitoring and treatment elements for (i) fever, (ii) hyperglycaemia (sugar) and (iii) swallowing protocols, and to the individual elements that make up each of these protocols.

The outcome for the economic evaluation will be the additional cost per quality-adjusted life year gained at 6 months post-stroke due to implementation of the FeSS Protocols. The process evaluation measure is the qualitative exploration of participants’ experience of contributing organisational, contextual and structural factors that impacted successful/unsuccessful uptake of the intervention.

### Sample size calculation

We used Monte Carlo simulation to estimate the detectable difference-in-difference between any two treatment arms with 80% power at a 5% significance level. Previous QASC Trial and QASC Europe studies suggest we could expect an ICC of 0.075, cluster level correlation between baseline and post-test of 0.3 and baseline defect-free care of 10%. With an assumed 60 hospitals with 60 patients included per hospital pre- and again 60 patients included post-intervention (i.e. *n*=120 patients per hospital and using an unequal randomisation ratio of 2 (high intensity):2 (low intensity):1 (no facilitation intensity), we calculated that a test between two 24 hospital arms would be able to detect a difference-in-difference of 1.5 (odds ratio (OR)) and a test between one 12 hospital arm and one 24 hospital arm would be able to detect a difference-in-difference of 2 (OR).

### Data analysis

#### Primary and secondary outcomes

The unit of analysis is at the patient level. Analysis will be by intention-to-treat. Demographic and clinical characteristics of patients (e.g. treatment in a stroke unit, discharge destination, ability to walk on discharge, duration of hospital stay, discharge modified Rankin Scale score) will be summarised using descriptive statistics and presented by group allocation. Analysis for overall adherence with all monitoring and treatment elements of the FeSS Protocols and adherence to each of the monitoring and treatment elements for Fever, Sugar and Swallowing will be undertaken based on the following comparisons: low-intensity facilitation versus control groups, high-intensity facilitation versus control groups and low-intensity facilitation versus high-intensity facilitation groups. Analysis will include comparison of outcomes between metropolitan and rural/remote hospitals; difference in proportions of change in monitoring and treatment for fever, hyperglycaemia and swallowing between stroke units versus stroke services. Analysis of outcomes will use logistic regression with adjustment for rurality, stroke unit presence; presence of Angels consultants; baseline thrombolysis rate (<11% vs ≥11%; 11% is the average annual rate based on the 2021 Stroke Foundation clinical audit) [5]; age, sex, stroke severity and correlation of outcomes within hospitals. Effect sizes will be estimated for each intervention group relative to the control as well as for comparisons between the low-intensity facilitation and high-intensity facilitation intervention groups. No interim analyses are planned.

#### Economic evaluation

Multiple imputation will be used to handle missing data used to estimate costs and quality-adjusted life years. Multivariable regression models will be used to adjust for differences in characteristics between control and intervention cohorts. Differences in costs and quality-adjusted life years gained between groups will be adjusted for confounding factors. Bootstrapping will be undertaken to test the robustness of results. Sensitivity analyses will also be performed to assess the effects of various assumptions in the economic modelling.

#### Process evaluation

Qualitative data from the process evaluation will be thematically analysed and will identify success factors and areas for strengthening the multicomponent implementation strategy for future use. Themes will be mapped to the Normalisation Process Theory to strengthen understanding of the reasons for why the different interventions may have worked [70].

## Discussion

In healthcare settings, facilitation aims to support a sustainable evidence-informed practice change based on an identified gap in clinical performance, with the goal of improving patient outcomes [72]. It ranges from providing task-oriented assistance to enabling individual clinicians and hospital units to alter their ways of thinking and working [73]. Our prior work in Europe has shown that an external remote facilitation approach can be successfully used for large-scale translation of the FeSS Protocols. Despite this evidence, the optimal intensity needed to effectively implement the Protocols into clinical practice remains unknown. This three-arm cluster randomised controlled trial will use a theory-informed behaviour change multicomponent implementation strategy to test the type of external remote facilitation needed to improve implementation of the FeSS Protocols for stroke patients in Australia and New Zealand. Stroke unit/stroke service clinicians will be supported to implement the FeSS Protocols in the context of a recognised need for improvement in stroke care practice.

For our pragmatic implementation trial [74], we have chosen the multi-arm (three-arm) parallel-group randomised trial design. Multi-arm randomised trials in general have the advantage of comparing multiple implementation strategies compared to two-arm trials [75, 76]. This is a key strength of our study as this design provides the opportunity to compare different doses of facilitation thereby increasing the possibility of detecting an effective dose.

Hospitals participating in this trial will be unequally allocated to the study arms in a 2:2:1 ratio, in favour of the intervention arms. This increases power, or decreases detectable difference at a given power, to compare pre-post changes in the high and low external remote facilitation intensity groups [77]. We expect increases to FeSS Protocol adherence to be greater in the intervention arms than in the control arm and our trial design will allow detection of smaller differences between the two active arms. In implementation research, interventions often operate at multiple levels and include health system changes [74]. Therefore, to reduce the potential for contamination in our trial, the unit of randomisation will be clusters (hospitals). An additional strength of our trial is that we will be recruiting hospitals in two countries and conducting a theory-based process evaluation to examine the mechanisms through which clinician behaviour change and implementation of the FeSS Protocols occurs. We will also be conducting an economic evaluation to make a business case for the benefits to healthcare organisations for investing in the uptake of the FeSS Protocols based on the most effective facilitation intervention identified.

Our trial has several limitations. Blinded outcome assessment is not feasible as data will be collected by hospital staff, some of whom may be involved in the study. Our trial is also using self-reported data from participating hospitals which may introduce reporting bias [78]. However, given that the trial outcomes are objective, the potential for reporting bias is minimal. In addition, self-auditing has similar overall error rates when compared with external auditing [79] and, importantly, supports the use of routinely collected hospital data by clinicians to drive evidence-based practice change. Another limitation is that all hospitals, including control group hospitals, possibly may be participating in other ongoing national stroke quality improvement initiatives (e.g. biennial national stroke acute care audits and/or AuSCR) run outside the trial and thereby receive routine feedback on their stroke care performance during the project. These initiatives potentially may improve control hospital FeSS performance. However, the potential for uncontrolled ‘noise’ from these basic reports is minimal, as the mechanism of feedback is passive.

Despite these limitations, our study is highly significant as it has the potential to accelerate the delivery of evidence-based acute stroke care in Australian and New Zealand hospitals by advancing knowledge of new effective models for implementation of the FeSS Protocols, while also examining fidelity of implementation and cost-effectiveness. The findings will demonstrate feasibility of large-scale translation of the Protocols by embedding their use in routine stroke care. In addition, we will generate a new theory-informed behaviour change FeSS Protocol implementation package with tools and instructions for implementation scale-up and spread. Our study also is an excellent example of using registry data to inform the quality of care. This package will be publicly available beyond this study and may be relevant to multiple stroke quality improvement initiatives.

Globally, stroke remains the second leading cause of death with the greatest burden experienced in lower- and middle-income countries where hospitals are resource-poor with reduced access to the latest stroke therapies including thrombolysis or clot retrieval services [80, 81]. Hence, determining the most effective external remote facilitation intensity to accelerate translation and implementation of the nurse-initiated FeSS Protocols into stroke practice across Australian and New Zealand acute care settings is likely to improve protocol uptake and quality of care for stroke patients on a large scale. Our trial will inform further upscale and spread of the FeSS Protocols into hospitals worldwide, in a future global FeSS implementation study.

## Supplementary Information

**Additional file 1.** SPIRIT 2013 Checklist: Recommended items to address in a clinical trial protocol and related documents.

**Additional file 2.** CONSORT 2010 checklist of information to include when reporting a cluster randomised trial

**Additional file 3.** The TIDieR (Template for Intervention Description and Replication) Checklist.

**Additional file 4.** Implementation strategies used in intervention and control groups, mapped against the Capability, Opportunity, Motivation - Behaviour (COM-B) model, Theoretical Domains Framework (TDF) and Behaviour Change Wheel intervention functions.

**Additional file 5.** QASC Australia videos - mapping to behaviour change techniques and the behaviour change wheel.

## Data Availability

Not applicable.

## Abbreviations

ARIA: Accessibility and Remoteness Index of Australia
COM-B: Capability, Opportunity, Motivation – Behaviour Model
FeSS: Fever, Sugar, Swallow
ICD-10: International Classification of Diseases, Tenth Revision
NSW: New South Wales
OR: Odds ratio
QASC: Quality in Acute Stroke Care
QASCIP: Quality in Acute Stroke Care Implementation Project
RES-Q: Registry of Stroke Care Quality
TDF: Theoretical Domains Framework
