# Supplementary material for: Evaluating remote facilitation intensity for multi-national translation of nurse-initiated stroke protocols (QASC Australasia): a protocol for a cluster randomised controlled trial
Source: Implement Sci. 2023 Jan 26;18:2. doi: 10.1186/s13012-023-01260-9 (PMC9879239; doi:10.1186/s13012-023-01260-9)
Supplement: Supplementary file 4 — Additional file 4. Implementation strategies used in intervention and control groups, mapped against the Capability, Opportunity, Motivation - Behaviour (COM-B) model, Theoretical Domains Framework (TDF) and Behaviour Change Wheel intervention functions. [file 13012_2023_1260_MOESM4_ESM.docx]

**Additional file 4: Implementation strategies used in intervention and control groups, mapped against the Capability, Opportunity, Motivation - Behaviour (COM-B) model, Theoretical Domains Framework (TDF) and Behaviour Change Wheel intervention functions**^21, 25^

| **COM-B Domain** | **TDF Domain** | 1. **Behaviour** 2. **change** 3. **intervention**   **function** | **Implementation strategies** | **High intensity facilitation** | **Low intensity facilitation** | **No facilitation** |
| --- | --- | --- | --- | --- | --- | --- |
| Psychological capability | Knowledge | Education | Online FeSS education package | 🗸 | 🗸 | X |
| Psychological capability | Behavioural regulation | Persuasion | Audit and feedback report | 🗸 | 🗸 | X |
| Psychological capability | Knowledge | Education | Education of stroke unit/stroke service clinicians | 🗸 | 🗸 | X |
| Psychological capability | Memory, Attention and Decision Processes | Enablement | Reminders* | 🗸 | 🗸 | 🗸 |
| Physical opportunity | Environmental Context and Resources | Enablement/Environmental  restructuring | Multidisciplinary workshops | 🗸 | 🗸 | X |
| Reflective motivation | Professional role and identity | Modelling | Use of clinical champions | 🗸 | 🗸 | 🗸 |
| Reflective motivation | Goals | Enablement | Videoconference sessions to support completion of FeSS education package, action plan development and FeSS Protocol implementation | 🗸 | X | X |
| Reflective motivation | Goals | Enablement | Email and telephone support to hospitals* | 🗸 | 🗸 | X |

*The intensity of reminders and email/telephone support differs between groups
