# Supplementary material for: Evaluating remote facilitation intensity for multi-national translation of nurse-initiated stroke protocols (QASC Australasia): a protocol for a cluster randomised controlled trial
Source: Implement Sci. 2023 Jan 26;18:2. doi: 10.1186/s13012-023-01260-9 (PMC9879239; doi:10.1186/s13012-023-01260-9)
Supplement: Supplementary file 5 — Additional file 5. QASC Australia videos - mapping to behaviour change techniques and the behaviour change wheel. [file 13012_2023_1260_MOESM5_ESM.docx]

# **Additional file 5: QASC Australia videos - mapping to behaviour change techniques and the behaviour change wheel**

1. **Fever is not the smoke it’s the fire video**

| **Behaviour Change Techniques** | **Comments/reasoning** | **Intervention Function** | **TDF** | **COM-B** |
| --- | --- | --- | --- | --- |
| 9.1 Credible source  (9 Comparison of outcomes) | information presented by SD and SM | Persuasion | Social Influences | Opportunity- Social |
| 4.1 Instruction on how to perform the behaviour  (4 Shaping knowledge) | SM outlines when to commence temperature check, when to start paracetamol and frequency of temperature check | Education | Knowledge | Capability- Psychological |
| 5.2 Salience of consequences  (5 natural consequences)  5.1 Information about health consequences  (5 natural consequences) | Nurse presents case study of consequences of not performing the target behaviour (Fe protocol)  SD presents information about health consequences for stroke recovery if not performed | Persuasion  Education | Beliefs about consequences  Knowledge | Motivation- reflective  Capability- Psychological |
| 5.2 Salience of consequences  (5 natural consequences) | Stroke survivor’s family member discussing personal experience associated with nursing staff conducting the behaviour | Persuasion | Beliefs about consequences | Motivation- reflective |
| 12.2 Restructuring the social environment  (12 Antecedents) | Information provided by nurse on how procedure was changed at her site to allow initial administration of paracetamol by nursing team | Enablement | Social influences | Opportunity- Social |
| 15.1 Verbal persuasion about capability  (15 self belief) | Nurse discussing story of successfully performing the target behaviour in practice | Modelling/ Persuasion | Social influences | Motivation- Reflective |

1. **FeSS cost effectiveness**

| **Behaviour Change Techniques** | **Comments/reasoning** | **Intervention Function** | **TDF** | **COM-B** |
| --- | --- | --- | --- | --- |
| 9.1 Credible source  (9 Comparison of outcomes) | Information presented by SD and SM  Frontline nurses sharing their experiences at beginning and end of video | Persuasion | Social Influences | Opportunity- Social |
| 15.1 Verbal persuasion about capability  (15 Self belief) | Nurses discussing story of having a positive impact on their practice of using the protocols | Modelling/ Persuasion | Social Influences | Motivation- Reflective |
| 5.1 Information about health consequences  (5. Natural consequences) | SD provides overview of health costs/benefits of implementing the protocols (e.g. reduced death and disability) | Education/Persuasion | Knowledge and  Beliefs about Consequences | Capability- Psychological  Motivation- Reflective |
| 5.1 Information about social consequences  (5. Natural consequences) | SM provides overview of societal costs/benefits of implementing the protocols | Persuasion | Beliefs about Consequences | Motivation- Reflective |
| 6.3 Information about others approval  (6 Comparison of behaviour) | Information provided that these protocols are recommended for use by the Stroke Foundation across Australia | Persuasion | Social Influences  Beliefs about capabilities  Professional role and identity | Opportunity- Social  Motivation- Reflective |
| 2.2 Feedback on behaviour  (2. Feedback and monitoring) | SM provides an overview on current rates of completion of components of FeSS protocols as per Australian National audit results | Persuasion | Beliefs about Consequences | Motivation- Reflective |

1. **FeSS is cost effective (Recruitment)**

| **Behaviour Change Techniques** | **Comments/reasoning** | **Intervention Function** | **TDF** | **COM-B** |
| --- | --- | --- | --- | --- |
| 9.1 Credible source  (9 Comparison of outcomes) | Information presented by SM and Charlene (RN) | Persuasion | Social Influences | Opportunity- Social |
| 5.1 Information about health consequences  (5 natural consequences) | SM discusses the impact of stroke on the community and globally and the benefits of implementing the FeSS protocols for patient outcomes (death and disability), healthcare costs and societal costs and their implementation globally, but that adherence still needs to be improved | Persuasion | Knowledge  Emotions | Capability- Psychological  Motivation- Reflective |
| 6.3 Information about others’ approval  (6. Comparison of behaviour) | SM references that the FeSS protocols are recommended in the Australian Clinical Guidelines for Stroke Management | Persuasion | Social Influences | Opportunity- Social |
| 15.1 Verbal persuasion about capability (15 Self belief) | SM describes how viewers can be part of improving care by signing up to be a study site | Persuasion/Enablement | Beliefs about capabilities | Motivation- Reflective |
| 5.2 Saliences of consequences (5 Natural consequences) | Charlene shares a story of successful implementation of the FeSS protocols at her site | Persuasion | Beliefs about consequences | Motivation- Reflective |

1. **Leading change can be hard**

| **Behaviour Change Techniques** | **Comments/reasoning** | **Intervention Function** | **TDF** | **COM-B** |
| --- | --- | --- | --- | --- |
| 9.1 Credible source  (9 Comparison of outcomes) | Story presented by Charlene (RN), instruction presented by credible source (Simeon Dale) | Persuasion | Social Influences | Opportunity- Social |
| 4.1 Instruction on how to perform the behaviour  (4 Shaping knowledge) | SD provides top tips on how to facilitate change within your organisation | Education/Enablement | Knowledge  Social Influences | Capability- Psychological  Opportunity- Social |
| 6.1 Demonstration of the behaviour  (6 Comparison of behaviour) | SM models with SD how to communicate when leading change | Modelling/Enablement | Social Influences/Beliefs about capabilities | Opportunity- Social  Motivation- Reflective |
| 13.1 Identification of self as role model  (13 Identity) | SD informs viewer that they are leader at their service and can be an example to others | Persuasion/Enablement | Social/Professional role and identity | Motivation- Reflective |
| 15.1 Verbal persuasion about capability (15 Self belief) | SD describes how viewers are leaders in their own service and know their team best to enact change | Persuasion/Enablement | Beliefs about capabilities | Motivation- Reflective |
| 5.2 Saliences of consequences (5 Natural consequences) | Charlene shares a story of successful implementation of the FeSS protocols at her site | Persuasion | Beliefs about consequences | Motivation- Reflective |

1. **A sip of water can be deadly**

| **Behaviour Change Techniques** | **Comments/reasoning** | **Intervention Function** | **TDF** | **COM-B** |
| --- | --- | --- | --- | --- |
| 9.1 Credible source  (9 Comparison of outcomes) | Information presented by Charlene (RN) and Emily (SP) | Persuasion | Social Influences | Opportunity- Social |
| 5.2 Salience of consequences  (5. Natural consequences) | Charlene describes the story of Mohammed who experienced aspiration pneumonia and subsequent complications as a consequence of having thin fluids prior to receiving a swallow screen  Family member describes the story of her sister, Linda receiving a swallowing screen by a nurse and how to prevented further complications after her stroke | Education/Persuasion | Beliefs about Consequences  Emotion | Motivation- Reflective  Motivation- Automatic |
| 5.1 Information about health consequences  (5. Natural consequences) | Emily (SP) discusses the incidence of swallowing problems after stroke and associated risks and the importance of nurses completing a swallow screening tool as recommended by their hospital | Education | Knowledge  Beliefs about consequences | Capability- Psychological  Motivation- Reflective |
| 4.1 Instruction on how to perform the behaviour  (4. Shaping knowledge) | Emily (SP) discusses the timeframe in which a swallow screen should be completed by nursing staff after stroke and where to access further training in use of the ASSIST tool | Education/Enablement/Training | Knowledge  Skills  Social influences | Capability- Psychological  Capability- Physical |

1. **Blood sugar is the fuel**

| **Behaviour Change Techniques** | **Comments/reasoning** | **Intervention Function** | **TDF** | **COM-B** |
| --- | --- | --- | --- | --- |
| 9.1 Credible source  (9 Comparison of outcomes) | Information presented by Charlene (RN) and Professor Cheung (endocrinologist) | Persuasion | Social Influences | Opportunity- Social |
| 5.2 Salience of consequences  (5. Natural consequences) | Charlene (RN) describes the story of Wei who experienced high BSL after stroke and had a poor outcome  Stroke survivor’s relative discusses her friend experiencing a poor outcome after stroke associated with delays in receiving insulin when there were experiencing high BSLs. | Education/Persuasion | Beliefs about Consequences  Emotion | Motivation- Reflective  Motivation- Automatic |
| Information about antecedents  (4. Shaping knowledge) | Professor Cheung discusses the incidence of hypoglycaemia after acute stroke | Education | Knowledge | Capability- Psychological |
| 5.1 Information about health consequences  (5. Natural consequences) | Professor Cheung discusses the increased risk of death and disability post stroke associated with high BSLs | Education | Knowledge  Beliefs about Consequences | Capability- Psychological  Motivation- Reflective |
| 4.1 Instruction on how to perform a behaviour  (4. Shaping knowledge) | Professor Cheung instructs on how often BSLs should be measured and when insulin should be given to reduce glucose levels | Education | Knowledge | Capability- Psychological |
| 6.3 Information about others’ approval  (6. Comparison of behaviour) | Professor Cheung references recommendations from the Australian Diabetes Society regarding the treatment of hyperglycaemia | Persuasion | Social Influences | Opportunity- Social |
| 1.4 Action planning  (1. Goals and planning) | Professor Cheung encourages viewer to check in at their hospital site on current protocols for insulin administration and to check in with their team about processes to ensure it is administered quickly and safely | Enablement | Beliefs about Capabilities | Motivation- Reflective |
| 12.5 Adding objects to the environment  (12. Antecedents) | Professor Cheung directs viewer to the insulin titration algorithm available for hyperglycaemia management if they do not have this resource available at their hospital | Enablement/environmental restructuring | Environmental context and resources | Opportunity- Physical |
| 15.1 Verbal persuasion about capability  (15 Self belief) | Charlene (RN) discusses making changes to procedures at her hospital and effectively implementing processes to support monitoring and management of hyperglycaemia | Modelling/ Persuasion | Social Influences | Motivation- Reflective |

1. **Why do an audit**

| **Behaviour Change Techniques** | **Comments/reasoning** | **Intervention Function** | **TDF** | **COM-B** |
| --- | --- | --- | --- | --- |
| 9.1 Credible source  (9 Comparison of outcomes) | Story presented by Charlene (RN), instruction presented by credible source (Prof Sandy Middleton, Simeon Dale) | Persuasion | Social Influences | Opportunity- Social |
| 4.1 Instruction on how to perform the behaviour  (4 Shaping knowledge) | SM and SD provides top tips on how to facilitate an audit and feedback session at your organisation | Education/Enablement | Knowledge  Social Influences | Capability- Psychological  Opportunity- Social |
| 15.1 Verbal persuasion about capability (15 Self belief) | SM and SD provide a summary of how conducting audit and feedback cycles can have a positive impact on practice | Persuasion/Enablement | Beliefs about capabilities | Motivation- Reflective |
| 5.2 Saliences of consequences (5 Natural consequences) | Charlene shares a story of successful using audit cycles at her site to improve adherence to the FeSS protocols | Persuasion | Beliefs about consequences | Motivation- Reflective |
